# Correction: Computational modeling of choice-induced preference change: A Reinforcement-Learning-based approach

**DOI:** 10.1371/journal.pone.0248442

**Published:** 2021-03-05

**Authors:** Jianhong Zhu, Junya Hashimoto, Kentaro Katahira, Makoto Hirakawa, Takashi Nakao

In the CBL models subsection of the Methods, there is an error in the fifth sentence of the first paragraph. The correct sentence is: α(0 ≦ α ≦ 1) is a parameter that determines the degree of updating of Q in one trial (learning rate).
