# Supplementary material for: Computational modeling of choice-induced preference change: A Reinforcement-Learning-based approach
Source: PLoS One. 2021 Jan 7;16(1):e0244434. doi: 10.1371/journal.pone.0244434 (PMC7790366; doi:10.1371/journal.pone.0244434)
Supplement: Attachment — Submitted filename: Response_To_reviewers.docx [file pone.0244434.s001.docx]

**Response to reviewers**

We are grateful for the excellent and extremely helpful comments. We addressed all the various issues, and we hope that the manuscript has now been improved considerably. Please let us know if further changes are necessary. We are more than happy to carry out such changes.

Modifications made to the main text and are shown using yellow highlights (see the separate version labeled “Manuscript with changes marked”).

Reviewer #1: General: Zhu et al., present results from a study of decision-making, in which they analyze internally guided decision-making within a reinforcement learning context called choice-based learning (CBL) to describe a phenomenon of internal decision making called choice-induced preference change (CPIC). At a high level, the theory of estimating the perceived rewards (Q value) starting from an initial arbitrary decision towards a goal of maintaining internal consistency is interesting, and if validated could provide an important consideration in analysis of decision-making. The specific simulation approach and parameter estimation function (fmincon) are outside of my area of expertise, and I’ll have to defer to experts in this area for specific commentary on the internal validity of the simulations performed. My main concern regarding the approach is that it is not clear to what extent the authors definitively ruled out prior external experience (value estimation) as biasing the initial choices. Other comments are given below.

*Thank you very much for your comments. We have responded to each comment in turn and indicated the changes made to the manuscript.*

Major:
-- Although the authors provide a solid explanation for how a CBL model for CPIC can be simulated using novel contour shapes, it is not clear to me how rigorous this approach is for avoiding differential initial preferences. In other words, how do we know that a subject does not have an initial preference for a given shape before the experiment? Is there a way to test this alternative possibility, or are we left to just assume based on intuition?

*This is very important. As the reviewer pointed out, it is impossible to confirm the exact initial values. When we collect the subjective rating of initial values of stimuli, it forms the preference before conducting a decision-making task. Besides, the subjective rating’s value is not the exact value since the latter is contaminated with noise associated with subjective evaluation (Izuma et al., 2013; Nakao et al., 2016, 2019). Therefore, even if the subjective rating’s values were used as an initial value in model analysis, it will eventually affect the estimated value of the model’s parameters. Hence, instead of collecting the initial subjective rating, we used novel contour shapes, which is widely accepted to assume that no prior preference has been formed in EDM studies. In the reward-based decision-making task, novel contour shapes are used, and their initial values are assumed to be 0, and the computational model has been applied. In the previous IDM studies, stimuli with obvious prior preferences (e.g., snacks) were used. To minimize the effect of the prior preferences, we used novel contour shapes by following EDM studies.*

*Nevertheless, as the reviewer suggested, we cannot completely rule out the possibility that there are differences in the stimulus’s initial values that may distort the parameter estimates. To achieve the first aim of examining the CBL model’s appropriateness, even if the effect of the initial value remains, we used the control model, which does not have the free parameters and predicts subsequent choice probabilities based on past choice history. The control model shares the assumption with the CBL model: the initial values of the stimuli are assumed equal. Hence, even then, the effect of the initial value difference cannot be completely ruled out by using novel contour shapes, a better fit of the CBL model to behavioral data than the control model indicates the CBL model’s appropriateness by incorporating free parameters such as learning rate and inverse temperature.*

*In the revised manuscript, we reflected the changes described above.*

*-------------------------------------------*

*Line 92*

The first aim of the present study was to examine the appropriateness of the CBL model in IDM by fitting the model to actual behavioral data. We applied the following two strategies to overcome the methodological limitation to applying computation analysis to IDM behavioral data. First, we used preference judgment for novel contour shapes as an IDM task. In EDM studies, novel contour shapes have been used, the initial value of which can be assumed to be equal, and applied to computational model analysis (e.g., [28-30]). By following the EDM studies, we used novel contour shapes to minimize the distortion of model parameter estimation caused by the different initial preferences. Second, we compared the CBL model with a control model without free parameters (e.g., learning rate) in which the probability of choosing an option was estimated based on the chosen frequency in the previous trials. The control model shared the assumption that there was no difference in initial preference among novel contour shapes in the CBL model and preference was estimated based on choice history. Hence, even if the effect of an initial value difference could not be completely ruled out by using novel contour shapes, a better fit of the CBL model to behavioral data than the control model indicated the CBL model’s appropriateness for incorporating free parameter.

*Line 161*

# Study 1

In Study 1, we examined (1) the CBL model’s appropriateness by using preference judgment with novel contour shapes and a control model, and (2) whether the value of both chosen and rejected items changed or not. The control model for the first aim estimated the probability of selecting an option based on the chosen frequency in the previous trials without free parameters. The control model shared with the CBL models the assumption that there is no difference in the initial value among the novel contour shapes.

*Line 275*

# Control model without free parameters

To examine the validity of setting the free parameters (i.e., $\alpha$ and *β*) in the CBL models, we conducted behavioral data analyses using a control condition without free parameters. In the control model, the value $V$of the chosen item *i* is given by

$V_{i}\left( t \right) =\frac{N_{i}^{chosen}(1:t-1)}{N_{i}^{presented}(1:t-1)}$	(4)

$N_{i}^{presented}(1:t-1)$is the number of times item *i* is presented before trial *t*. $N_{i}^{chosen}\left( 1:t-1 \right)$is the number of times item *i* is chosen among them. $V_{i}$ is 0 when the item *i* is presented for the first time.

The probability that the model selects the option chosen by a participant ($P_{chosen}$) is given by

$P_{chosen}=\frac{V_{chosen}\left( t \right)+1}{V_{chosen}\left( t \right)+V_{rejected}\left( t \right)+2}$	(5)

To define the probability, even if both the chosen and rejected items’ values are zero, one and two were added to the numerator and the denominator, respectively (see, Ito and Doya [37]).

*Line 304*

In this study, we used novel contour shapes to assume the initial values of stimuli are consistent when applying computational model analysis. We, therefore, set the initial value for all stimuli to 0.5 in the CBL models. The initial value of all items in the control model was set to 0 since the number of times all items presented was 0.

*Line 518*

Regarding the first aim, CBL models showed a better fit to IDM behavioral data than the control model (Fig 4). Although the results of Simulation 2 (Fig 3) showed that the control model was the best fit for a certain proportion of data even when the CBL (especially, CBL*α_c_* and CBL*α_r_*) models were the true model, the CBL model did not have the best fit when the control model was the true model. Since the CBL models had a better fit than the control model to the actual behavioral data, it was unlikely that the control model was the true model. The control and CBL models similarly assumed no difference in initial preference among novel contour shapes and estimated value based on the preference choice history. Since the main difference was the presence of free parameters (i.e., learning rate and reverse temperature), the better fit of the CBL models to behavioral data than the control model confirmed the CBL model’s appropriateness for incorporating free parameters.

*-------------------------------------------*

-- Figure 1 indicates that preference to images is provided using a Likert scale, although the computational models use Softmax function (actually it looks more like a logistic function) to calculate probabilities, and based on the description provided, choices are dichotomized. Was the ordinal relationship within the Likert scale addressed, and if not, why was this scale used in the experiment rather than a binary decision?

*Thank you for your comment. In the computational model analysis, we used the time series of binary preference choice data. Subjective preference ratings on the Likert scale were not used in the computational model analyses. The rating data were used to compare each item’s value, estimated from the binary preference judgment data.*

*In the original paper, the purpose of collecting Likert scale data and how to use it was unclear. In the revised paper, we described the intention to collect subjective ratings and explained that the rating data was not used in the computational model analyses. Please see the following:*

*-------------------------------------------*

*Line 128*

The second aim was to investigate whether the value of both chosen and rejected items changed without using subjective ratings. To achieve this aim, we compared the CBL model with both values changed and with the models with either the value of chosen or rejected item changed. Note that although we collected subjective preference ratings for all novel contour shapes after the IDM (i.e., preference choice) task, the rating data were not for the computational model analysis to examine the CIPC, but to examine the inconsistency between subjective ratings and choice behavior suggested by the previous study [31].

*Line 227*

In order to examine the consistency of preference judgment and subjective rating, the participants conducted the subjective preference rating task after the preference judgment task. In the rating task, participants were instructed to evaluate their subjective preference for each of the 15 novel contour shapes, rated on a 5-point scale (1 = *extremely dislike*, 5 = *extremely like*). The data of the preference ratings of the Likert scale were not used in the computational model analysis.

*Line 234*

**Fig 1. Experimental procedure of IDM task and subjective rating.** A) In the IDM task, participants were asked to choose his/her preferred option from two stimuli. The stimuli display time was 2,000 ms. In case participants did not respond during the period, the screen displayed a blank screen until the response. B) In a subjective preference rating, participants subjectively evaluated all 15 stimuli on a 5-point scale (1 = *extremely dislike*, 5 = *extremely like*). The data of the subjective rating were not used for the computational model analysis.

*-------------------------------------------*

-- Did the authors address autocorrelation within an individual’s selections? It would seem that the time between preference choices would be correlated, although it is not clear whether any of the models accounts for temporal autocorrelation, as for example, a hidden Markov model might.

*Thank you for your comment. As the reviewer pointed out,* *the CIPC is thought to cause autocorrelation in IDM. The CBL model is one of the computational models representing the autocorrelation as a value change in IDM: the chosen stimulus’s value increases and is more likely to be chosen later. Although the CBL model reflects a stimulus-based autocorrelation, this does not capture the trial-based temporal autocorrelation since only 2 out of 15 stimuli were presented in each trial.*

*Therefore, we set up an F-CBL that included an attenuation of the value of stimuli not presented. This model will be the best fit for a trial-based temporal autocorrelation in the IDM so that the stimulus chosen in the recent past is more likely to be chosen. Although we examined the trial-based temporal autocorrelation in IDM by comparing this CBL model with the F-CBL model, F-CBL did not show the best fit to the behavioral data.* *However, the parameter recovery of F-CBL was not as good as the simple CBL models examined in Study 1 in the revised manuscript. That is, it remains possible that the experimental paradigm of the present study was not designed well enough to validate the two modified CBL models. We described this point as the limitation.*

*Regarding the Markov model, Ito and Doya (2009) applied the Markov model to their EDM behavioral data and compared it with the RL models. We attempted to apply the Markov model to our IDM data. Unfortunately, unlike the case of Ito and Doya, it was impossible to estimate the state transition probability because the combination of stimuli presented differed in every trial in our IDM task: That is, participants experienced the same state only once. Hence, instead of applying the Markov model, we included the analyses described above.*

*We added the following descriptions in the revised manuscript.*

*-------------------------------------------*

*Line 137*

# Improvements in CBL-based models

In EDM studies, the RL models incorporating various parameters, such as reducing decision noise with the accumulation of experience in decision-making [32] and value forgetting over time [33], have been proposed. With reference to those EDM studies, as the third aim of the present study, we explored the possible modification of the CBL model to explain CIPC better.

*Line 575*

The F-CBL model was developed to explore the possibility that the value of the items not presented decayed over time. In the CBL models in Study 1, the values of the chosen and the rejected stimuli updated, while the value of the stimulus that was not present in a trial remained unchanged. To examine the possibility of attenuations of the values of unpresented stimuli, we added the forgetting factor ($\alpha_{F}$) to the CBL*ɑ_cr_* model.

The examination of the F-CBL model corresponded to the examination of trial-based temporal autocorrelation in IDM. The CIPC was considered to cause autocorrelation in IDM: chosen items’ preference increased and items were subsequently more likely to be chosen, while rejected items’ preference decreased and items were subsequently less likely to be chosen. The CBL model was one of the computational models that represented such autocorrelation. The CIPC was reflected in the time series behavioral data as a stimulus-based autocorrelation because two out of 15 stimuli were presented in the present study task and that could be captured by CBL*ɑ_cr_* model. However, it was also possible that the item chosen in the recent past was more likely to be chosen, that is, a trial-based temporal autocorrelation. Since the F-CBL included attenuation of the value of stimuli not presented in each trial, this model would be the best fit if there were a trial-based temporal autocorrelation in the IDM.

*Line 602*

*F-CBL model:* In this model, the value *V* of the items *i* that did not presented in each trial *t* was updated as follows:

$V_{i}\left( t+1 \right) ={\left( 1-\alpha_{F} \right)\times V}_{i}\left( t \right)$	$\mathrm{if}i was not presented$	(10)

where $\alpha_{F}$ is the forgetting factor which modulates the degree of attenuation of the value. The forgetting factor was included by following the previous EDM study [33]. In this model, the forgetting factor was applied to unchosen items, unlike the present study.

*Line 670*

In the first place, however, it should be noted that the parameter recovery of the T$\beta$-CBL and F-CBL models was not as good as that of the CBL*α_cr_*. That is, it remains possible that the experimental paradigm of the present study was not designed well enough to validate the two modified CBL models. For example, looking at the results of parameter recovery for the F-CBL model (Fig 7), which was prepared to examine trial-based temporal autocorrelation, adding the forgetting factor *α_F_* in the CBL*α_cr_* model reduced the accuracy of *α_cr_* estimation (c.f., Fig 2). The learning rate *α_cr_* led to a stimulus-based autocorrelation such that the chosen item was more likely to be chosen when presented later. Theoretically, in the present paradigm, the same item was presented every seven trials on average. Hence, it is possible that stimulus-based autocorrelation and trial-based autocorrelation are not easily estimated separately. Future experiments with an increased number of items may be useful to evaluate the F-CBL model properly.

*Line 697*

The third aim was to explore the possible modification of the CBL model. From the computational model analysis of the behavioral data in Study 2, the $\mathrm{CBL}\alpha_{cr}$model fitted better than the other modified models, T*β*-CBL and F-CBL models. However, the parameter recovery of the two modified models was not as good as the $\mathrm{CBL}\alpha_{cr}$model and there remained the possibility that the present study’s paradigm was not appropriate for considering these modifications.

Taken together, it was shown that the CBL$\alpha_{cr}$ model (and also potentially CBL$\alpha_{c}\alpha_{r},$see Discussion in Study 1) could explain CIPC in IDM, the potential for further model improvement remains, though.

*Line 751*

Third, as we discussed in Study 2, to examine the validity of the modified CBL model (i.e., TB-CBL and F-CBL), it is desirable to conduct experiments with appropriate task design to test those models.

*-------------------------------------------*

-- Since PLOS One is aimed toward more general readership, the authors might consider widening the scope with more explanation about the applications of this approach within the field of neurology/psychology and behavior.

*Thank you for your suggestion. Based on the reviewer’s comment, we added the following descriptions to the Discussion section.*

*-------------------------------------------*

*Line 755*

# Conclusion

In this study, we carried out the preference judgment task with novel contour shapes to apply computational modeling to IDM. From simulations and a behavioral experiment with computational modeling as with EDM, we showed that the CBL model could describe CIPC, and we confirmed that the value of both the chosen and rejected items change without using subjective ratings. The computational modeling allowed us to estimate trial-by-trial preference change and model parameters (e.g., learning rate). Those parameters could apply for further analyses, such as for neural bases of IDM, as had been done in EDM research. That would lead to an increase in an integrated understanding of EDM and IDM; for example, whether learning rates, which have common psychological meanings for EDM and IDM, are common in the neural substrates.

*-------------------------------------------*

Minor:
-- Perhaps it reflects my distance from the subject matter, but the initial statement ‘the value of an item is learned through making a series of decisions,’ (line 45) seems fairly abstract and no further information is given to support what context that value is made? What ‘item’ is being referred to? A decision tool? An item of value being purchased? As above, some additional context in the Background could be helpful.

*“Item” here refers to something we chose after decision-making. We learned the value of these items through these decisions or actions. However, as the reviewer pointed out, “item” in the original paper was not clearly defined and prone to misunderstanding. Therefore, we deleted this sentence in the manuscript and changed the beginning to “The value learning process used by humans and animals has been investigated using a decision-making task with a correct answer specified by the external environment (externally guided decision-making, EDM; [1]).”*

-- In most RL applications, the learning rate is a hyperparameter, and thus is assigned outside the modeling process. On line 56, the authors note that the learning rate is estimated as a model parameter. If there is another method the authors are applying to identify a learning rate, it should be provided, otherwise this section should be corrected to indicate that LR is a hyperparameter, and must be optimized through manual or automated search (e.g., grid search), not fit like a statistical parameter. If this optimization is all performed by fmincon function, then more information about the specific algorithm is needed for us less informed readers.

*In the context of machine learning, as the reviewer described, the learning rate was set as a hyperparameter, while in the context of computational modeling of behavioral data, the learning rate was treated as an inferable parameter (e.g., Sugrue et al., 2004; Ito & Doya, 2009).*

*Here, we used the maximum-likelihood approach to estimate the parameters that best describe behavioral data, which is the broadly used method in decision-making studies. The log-likelihood (LL) for all trials was written in terms of the choice probabilities of the individual model for the chosen option in behavioral data as*

$LL=\sum_{t=1}^{T} logP_{chosen}(t)$

*where T represents the total number of trials. The* $P_{chosen}$ *is the probability that the model selects the stimuli actually chosen by a participant in each trial t, calculated based on the formulas (2) and (3) in the main text. We used Matlab’s fmincon function* *with an interior-point method to estimate the learning rate (α) and the gradient of the softmax function (β) that maximizes the likelihood of each model generating behavioral data (for the interior point algorithm, please refer to the research of Yin Zhang (1998).*

*(Yin Zhang (1998) Solving large-scale linear programs by interior-point methods under the Matlab Environment, Optimization Methods and Software, 10:1, 1-31, DOI: 10.1080/10556789808805699).*

*In response to the reviewer’s comment, we added these explanations to the revised manuscript.*

*-------------------------------------------*

*Line 314*

We estimated the best fitting parameters for each artificial behavioral dataset by the maximum likelihood method. The log-likelihood (*LL*) for all the *trials* was written in terms of the choice probabilities of the individual model for the chosen option in the behavioral data as

$LL=\sum_{t=1}^{T} \log P_{chosen}(t)$	(6)

where *T* represents the total number of trials. The $P_{chosen}$ is the probability that the model selects the stimuli actually chosen by a participant in each trial *t*, calculated based on formulas (2) and (3). We used Matlab’s fmincon function with an interior-point method [38] to estimate the free parameters that maximize the likelihood of each model generating behavioral data. Optimality tolerance and step tolerance were set to 1e-8. The search range of *α* and $\beta$ were the same as the range set during artificial data generation.

*Line 328*

We conducted Simulation 2 (model recovery) to confirm whether the true model showed the best fit to the behavioral data generated from that model. *To* evaluate the relative goodness of fit of the models, the AIC (Akaike Information Criterion) was used, which is given by

$\mathrm{AIC}= -2LL+2k$	(7)

where *k* is the number of free parameters. A smaller AIC denotes a better model fit to the data.

*-------------------------------------------*

-- Table 2 is referenced before Table 1

*Thank you for your suggestion. We moved Table 2 to the introduction of Study 1 in the revised manuscript.*

-- How was the range of B of [0, 20] chosen?

*Thank you for your question. We set the β in that range because a larger value of β generates random decision-making in the artificial data. The data does not reflect the differences between the models. In fact, parameter recovery did not work well in a large range, 0 < β < 100. However, to deal with the possibility that the actual value of β is greater than 20, we also performed an additional analysis with the search range of β set to 0-100. We found that the result was the same as in the original manuscript with the range of β in the range of (0, 20).*

*We added the explanation about this point and also reported the results with the search range, 0 < β < 100.*

*-------------------------------------------*

*Line 308*

The range of parameters set for each model when generating artificial behavioral datasets is shown in Table 3. “$U$” indicates a uniform distribution, and the value of *α* was randomly selected from a uniform distribution on the interval (0, 1), and the value of $\beta$ was randomly selected from a uniform distribution on the interval (0, 20). We set *β* in that range because a larger value of *β* generates random choice behavioral data, which reflects the differences of the models less.

*Line 341*

# CBL model fit to the behavioral data

We conducted computational model analyses of the actual behavioral data. The method of model parameter estimation was the same as Simulations 1 and 2. However, to deal with the possibility that the actual value of *β* could be greater than 20, we performed an additional analysis with the search range of *β* set to 0–100.

Line *464*

In cases where the search range of *β* was set to 0–100, similar results were observed to when the search range was 0–20, except that no significance was found between CBL$\alpha_{cr}$ and CBL*α_c_α_r_*. The mean AICs for CBL$\alpha_{c}$, CBL$\alpha_{r}$, CBL$\alpha_{cr}$, CBL*α_c_α_r_*, and the control models were 121.49 (*SE*=2.40), 120.28 (*SE*=2.48), 115.14 (*SE*=2.71), 115.71 (*SE*=2.69), and 149.16 (*SE*=0.71), respectively. All the CBL models showed better fits to the behavioral data than the control model (*t*s(47) > 11.23, *p*s < .001; Holm). CBL$\alpha_{cr}$ (*t*s(47) > 6.14, *p*s < .001; Holm) and CBL*α_c_α_r_* (*t*s(47) > 5.98, *p*s < .001; Holm) showed better fits than the other models.

*-------------------------------------------*

Reviewer #2: The paper presents validity of the CBL model that has not been previously confirmed by fitting the model to IDM behavioral data, since the differences of initial preferences among the items make it difficult to estimate the model parameters. Hence, the paper presents a set of experiments conducted with novel contour shapes that are supposed to be equaly probably selected.
The paper is very well writen and explain clearly the main concepts. In addition, the discussion part has very clear and concise arguments that support the experiments results.

Figure 2 should be created with more definition as it is blurred when the size is big.

*Thank you very much for your comments.*

*The dpi of the original version of Figure 2 was 150. We enhanced it to 450 dpi in the revised manuscript.*

Reviewer #3: In this study, the authors suggested the validity of the choice-based learning model in internally guided decision-making (IDM). They first tested whether the model parameters are estimated by fitting the model to the artificial data. They then confirmed whether models can explain the artificial data. They finally verified the computational model by fitting the model to behavior choice data.

1

The authors reported that the CBL model fit the IDM behavioral data well.

The authors only showed that Model 3 (CBL model) is the best fit for the data among 3 models. They did not show how much Model fits the behavioral data.

I would recommend showing the model fit the data by giving an example, and/or by using some indicators.

*Thank you for your comment.*

*The log-likelihood (LL), which is used to calculate AIC, is the sum of the probability that the model will actually select the selected stimulus across trials. However, as the reviewer suggested, the AIC is not an indicator that brings an intuitive understanding of the extent of the model fit behavior. Hence, from LL, we calculated the normalized likelihood which is the averaged probability per trial of the model selecting the item actually chosen by a participant. However, different from the AIC, the normalized likelihood is not adjusted for the impact of the number of free parameters.*

*Furthermore, we noticed we did not report the equations to calculate LL and AIC.*

*We added the following to the revised manuscript.*

*-------------------------------------------*

*Line 314*

We estimated the best fitting parameters for each artificial behavioral dataset by the maximum likelihood method. The log-likelihood (*LL*) for all the *trials* was written in terms of the choice probabilities of the individual model for the chosen option in the behavioral data as

$LL=\sum_{t=1}^{T} \log P_{chosen}(t)$	(6)

where *T* represents the total number of trials. The $P_{chosen}$ is the probability that the model selects the stimuli actually chosen by a participant in each trial *t*, calculated based on formulas (2) and (3). We used Matlab’s fmincon function with an interior-point method [38] to estimate the free parameters that maximize the likelihood of each model generating behavioral data. Optimality tolerance and step tolerance were set to 1e-8. The search range of *α* and $\beta$ were the same as the range set during artificial data generation.

*Line 328*

We conducted Simulation 2 (model recovery) to confirm whether the true model showed the best fit to the behavioral data generated from that model. To evaluate the relative goodness of fit of the models, the AIC (Akaike Information Criterion) was used, which is given by

$\mathrm{AIC}= -2LL+2k$	(7)

where *k* is the number of free parameters. A smaller AIC denotes a better model fit to the data.

*Line 341*

# CBL model fit to the behavioral data

We conducted computational model analyses of the actual behavioral data. The method of model parameter estimation was the same as Simulations 1 and 2. However, to deal with the possibility that the actual value of *β* could be greater than 20, we performed an additional analysis with the search range of *β* set to 0–100.The AICs were calculated using all the models that fulfilled the criteria in the two simulations by applying the choice data of each participant to the model. The AICs were tested using the multiple-comparison Holm method, adjusted for all possible combinations of the model comparisons.

For an intuitive understanding of how much the models predicted the behavioral data, we calculated the normalized likelihood [37] given by

$z_{L}=e^{(LL/T)}$	(8)

where *LL* is the log-likelihood calculated based on formula (6), and *T* represents the total number of trials. The normalized likelihood represents the averaged probability per trial of the model selecting the item actually chosen by a participant. This index presents 0.5 when the probability of the model choosing the actually chosen item by a participant is a chance-level. A larger normalized likelihood denotes better model prediction to the behavioral data. However, different from the AIC, the normalized likelihood was not adjusted for the impact of the number of free parameters.

*Line 456*

The normalized likelihood for CBL$\alpha_{c}$, CBL$\alpha_{r}$, CBL$\alpha_{cr}$, CBL*α_c_α_r_*, and the control models were 57.17% (*SE*=0.64), 57.55% (*SE*=0.67), 59.08% (*SE*=0.75), 59.47% (*SE*=0.74), and 49.16% (*SE*=0.16), respectively. Similar to the results of the AIC, all the CBL models showed higher probability to select the actually chosen option than the control model (*t*s(47) > 12.21, *p*s < .001; Holm). CBL*α_cr_* showed a significantly higher probability than the other models (*t*s(47) > 6.37, *p*s < .001; Holm), except CBL*α_c_α_r_*. CBL*α_c_α_r_* showed a significantly higher probability than CBL*α_cr_* (*t*(47) = 5.39, *p* < .001; Holm).

*Line 651*

The normalized likelihoods for CBL*α_cr_*, T$\beta$-CBL, and F-CBL were 59.08% (*SE* = 0.75), 54.04% (*SE* = 0.26), and 59.20% (*SE* = 0.75), respectively. CBL*α_cr_* and F-CBL showed higher probability to select the actually chosen option than T$\beta$-CBL (*t*s(47) > 9.34, *p*s < .001; Holm). F-CBL showed a higher probability than CBL*α_cr_* (*t*(47) = 2.27, *p* = .028; Holm).

*-------------------------------------------*

2

There are other models to explain choice behavior. For example, Ito and Doya showed the 3rd Markov model and RL model with the time-varying parameters were the better descriptive model of rats’ choice strategies than the F-Q model with constant parameters (corresponding to Model 3). I would suggest testing models with time-varying parameter (and Markov model).

（Ito M and Doya K. Validation of decision-making models and analysis of decision variables in the rat basal ganglia. J Neurosci (2009) 29:9861–9874. doi:10.1523/JNEUROSCI.6157-08.2009）

*Thank you for your comments. Regarding the Markov model, as the reviewer pointed out, Ito and Doya (2009) applied the Markov model to their EDM behavioral data and compared it with the RL models. We attempted to apply the Markov model to our IDM data. Unfortunately, unlike the case of Ito and Doya, it was impossible to estimate the state transition probability because the combination of stimuli presented differed in every trial in our IDM task: That is, participants experienced the same state only once. Hence, instead of applying the Markov model, we added the following three models.*

*Control model: This model had no free parameters but shared the assumption with the CBL models that no difference in initial preference among novel contour shapes and the preference is estimated based on choice history. This model was introduced as a response to reviewer #1’s comment.*

*Tβ-CBL (CBLɑcr model with time-varying* $\beta$*): this model was constructed to examine the possibility that the more a stimulus is experienced, the less noise is included in the IDM by following the suggestions from reviewer #3 and #4.*

*F-CBL: We added the F-CBL model which is similar to the F-Q model proposed by the reviewers. This model was developed to explore the possibility that the unpresented items’ value decayed over time. The examination of the F-CBL model corresponds to the examination of trial based temporal autocorrelation in IDM. This model was introduced as a response to reviewer #3 and #1’s comments.*

*-------------------------------------------*

*Line 100*

Second, we compared the CBL model with a control model without free parameters (e.g., learning rate) in which the probability of choosing an option was estimated based on the chosen frequency in the previous trials. The control model shared the assumption that there was no difference in initial preference among novel contour shapes in the CBL model and preference was estimated based on choice history. Hence, even if the effect of an initial value difference could not be completely ruled out by using novel contour shapes, a better fit of the CBL model to behavioral data than the control model indicated the CBL model’s appropriateness for incorporating free parameter.

*Line 566*

# Study 2

In Study 2, we did an exploratory investigation into the possible modification of the CBL model to explain CIPC better. We constructed and validate modified T*β*-CBL (CBL*ɑ_cr_* model with time-varying $\beta$) and F-CBL (CBL*ɑ_cr_* model with the forgetting parameter for unpresented items) based on the CBL*ɑ_cr_* model that had a best fit to the behavioral data in Study 1.

The T*β*-CBL was constructed to examine the possibility that the more a stimulus was experienced, the less noise was included in the IDM. To investigate this possibility, we set th*e β* as a variable over time by following Yechiam et al. [32].

The F-CBL model was developed to explore the possibility that the value of the items not presented decayed over time. In the CBL models in Study 1, the values of the chosen and the rejected stimuli updated, while the value of the stimulus that was not present in a trial remained unchanged. To examine the possibility of attenuations of the values of unpresented stimuli, we added the forgetting factor ($\alpha_{F}$) to the CBL*ɑ_cr_* model.

The examination of the F-CBL model corresponded to the examination of trial-based temporal autocorrelation in IDM. The CIPC was considered to cause autocorrelation in IDM: chosen items’ preference increased and items were subsequently more likely to be chosen, while rejected items’ preference decreased and items were subsequently less likely to be chosen. The CBL model was one of the computational models that represented such autocorrelation. The CIPC was reflected in the time series behavioral data as a stimulus-based autocorrelation because two out of 15 stimuli were presented in the present study task and that could be captured by CBL*ɑ_cr_* model. However, it was also possible that the item chosen in the recent past was more likely to be chosen, that is, a trial-based temporal autocorrelation. Since the F-CBL included attenuation of the value of stimuli not presented in each trial, this model would be the best fit if there were a trial-based temporal autocorrelation in the IDM.

*-------------------------------------------*

3

The data points are unclear in Figures 5 and 6. I would suggest showing all the data points. I also suggest showing the correlation between the degree of conflict and RT for Figure 5, and the correlation between preference rating and chosen frequency for Figure 6

*Thank you for your comments. In response to the reviewer’s comment, data points are added to Fig 4, 5, 6 and 9 in the revised manuscript.*

*We also conducted correlation analyses. The Pearson’s correlation coefficient between RT and the difference of chosen frequency as the index of conflict was calculated after outliers (from the mean ± 3SD) was excluded in each participant. The r values were transformed to Fisher’s Z. One sample t-test for the Z value against 0 showed the correlation was significant. We reported the results in the revised manuscript and the scatter plot of all the trials’ data from all participants were added in Figure 5.*

*Regarding the correlation between preference rating and chosen frequency, we also conducted the same correlation analysis, and no significant correlation was found. We reported these results and a scatter plot of data from all participants’ trials in Fig 6 of the revised manuscript.*

*-------------------------------------------*

*Line 452*

**Fig 4. AIC results after fitting each model to actual behavioral data (Study 1).** * *p* < .05, ** *p* < .001. The black dots, error bars, and colored dots indicate the average, standard deviation (*SD*), and each participant’s data, respectively.

*Line 487*

**Fig 5. The relationships between the degree of conflict and reaction time (RT).** (a) The mean average of RT in high- and low-conflict conditions (back dots), *SD*, and each participant’s data (colored dots). ** *p* < .001. (b) A scatter plot of all the trial data of all the participants between RT and the difference of chosen frequency. The trials with a larger difference in the chosen frequency corresponded to those with less conflict. The black regression line corresponds with all the data points of all the participants. The orange and green dots and the regression lines correspond with the participants’ data that showed the strongest negative correlation and the data that did not, respectively.

*Line 505*

**Fig 6. The relationship between subjective preference rating and chosen frequency in a preference judgment task.** (a) Mean subjective preference ratings for frequently chosen (high-frequency) and less chosen (low-frequency) stimuli (back dots), *SD* (error bars), and each participant’s data (colored dots). (b) A scatter plot of all stimulus data from all participants between subjective preference rating and chosen frequency. The black regression line corresponds to all data points of all participants. The orange and green dots and regression lines corresponding to participant’s data showed the strongest negative correlation and those that did not, respectively.

*Line 647*

**Fig 9. AIC results after fitting each model to actual behavioral data (Study 2).** ** *p* < .001. The black dots, error bars, and colored dots indicate the average, *SD*, and each participant’s data, respectively.

*-------------------------------------------*

4

One of the CBR models (model 4) failed to fit the data. There is a lack of explanation of why Model 4 failed to fit the data from Model 4 in Figure 3 even the parameter recovery of Model 4 succeeded in Figure 2.

*Thank you for your comments. As you pointed out, the explanation was not enough. We added the explanation about this point in the revised manuscript.*

*In relation to this point, in the actual behavioral data analyses, some participants showed a best fit to Model 1 or 2 (those models are named CBL*$\alpha_{c}$ *and CBL*$\alpha_{r}$*, respectively, in the revised manuscript). Taken together with the fact that Model 4 (CBL*${\alpha_{c}\alpha}_{r}$*) was successful in parameter recovery, CBL*${\alpha_{c}\alpha}_{r}$ *is likely to be appropriate for parameter estimation in individual participants. We also discussed this point in the Discussion section.*

*-------------------------------------------*

*Line 428*

One plausible reason for the failure of the CBL*α_c_α_r_* model recovery is that in the cases the randomly selected *α_c_* and *α_r_* to generate the artificial data using CBL*α_c_α_r_* were similar to each other, CBL*α_cr_* fitted the artificial data. Meanwhile, CBL*α_c_* or CBL*α_r_* fitted best when there was a large difference in the learning rates. In fact, as we can see in Fig 3, compared with the cases where the CBL*α_cr_* model generated the artificial data, there are more cases where CBL*α_c_* or CBL*α_r_* provided the best fit when CBL*α_c_α_r_* generated the artificial data. Besides, as shown in Table 4, the difference between *α_c_* and *α_r_* to generate the artificial data by CBL*α_c_α_r_* was larger in the cases the CBL*α_c_* or CBL*α_r_* adopted than the case of the CBL*α_cr_* was the best.

**Table 4. Summary of randomly determined learning rates to generate artificial data using CBLαcαr in the cases where the other three CBL models showed the best fit (Simulations 2).**

| **The best fit model** | **Proportion of artificial data**  **where *α_c_* > *α_r_*** | **Mean *α_c_*** | **Mean *α_r_*** |
| --- | --- | --- | --- |
| CBL$\alpha_{c}$ | 100 % | 0.58 | 0.07 |
| CBL$\alpha_{r}$ | 0% | 0.03 | 0.60 |
| CBL$\alpha_{cr}$ | 50% | 0.52 | 0.53 |

*Line 538*

Importantly, although CBL*α_cr_* was adopted in model comparisons, there remained the possibility that the CBL*α_c_α_r_* was a true model and that was likely useful for parameter estimation of individual learning. Although CBL*α_c_α_r_* had failed model recovery in Simulation 2, as can be seen in Fig 3, the results of this model recovery indicated that the true model could be CBL*α_c_α_r_* when CBL*α_cr_* was the best fit. Besides, in Simulation 1, the CBL*α_c_α_r_* model showed good parameter recovery. Furthermore, although averaged AIC by fitting the actual participants’ data was better in CBL*α_cr_* than in CBL*α_c_*, CBL*α_r_*, and CBL*α_c_α_r_* (Fig 4), when we compared AIC in an individual participant’s data, 16.67%, 10.42 %, and 10.42 % of participants showed a best fit to CBL*α_c_*, CBL*α_r_* and CBL*α_c_α_r_*, respectively. For those participants’ data, the estimating parameters in CBL*α_cr_* were likely to be inappropriate. For parameter estimation in individual participants, including those who had different learning rates for chosen and rejected items, it was possible that applying CBL*α_c_α_r_*, which is a model that encompasses the CBL*α_c_* and CBL*α_r_*, that CBL*α_cr_* would be plausible.

*-------------------------------------------*

5

The authors did not clearly state which statistical test they used. It is also unclear whether they used multiple comparison correction in Figure 4.

*This is an important point. We applied Holm’s multiple comparison correction. We added the description in the manuscript as follows.*

*-------------------------------------------*

*Line 347*

The AICs were tested using the multiple-comparison Holm method, adjusted for all possible combinations of the model comparisons.

*Line 444*

Fig 4 shows a comparison between the models where the search range for alpha and beta were 0–1 and 0–20, respectively. All the CBL models showed a better fit to the behavioral data than the control model (*t*s(47) > 11.30, *p*s < .001; Holm). CBL$\alpha_{cr}$ showed the best fit for the behavior data (*t*s(47) > 2.47, *p*s < .035; Holm).

*-------------------------------------------*

6

There are only a few descriptions of the figures. Especially, there is no information on the numbers in the matrix in Figure 3.

*Thank you for your comments. We added a detailed description to the figures’ captions.*

*-------------------------------------------*

*Line 234*

**Fig 1. Experimental procedure of IDM task and subjective rating.** A) In the IDM task, participants were asked to choose his/her preferred option from two stimuli. The stimuli display time was 2,000 ms. In case participants did not respond during the period, the screen displayed a blank screen until the response. B) In a subjective preference rating, participants subjectively evaluated all 15 stimuli on a 5-point scale (1 = *extremely dislike*, 5 = *extremely like*). The data of the subjective rating were not used for the computational model analysis.

*Line 399*

**Fig 2. Results of parameter recovery in Study 1 (Simulation 1).** A parameter recovery was conducted to confirm whether each model satisfied estimating the model parameters from the choice data. The correlation coefficients between the parameters used to generate the artificial data are shown as parameter recovery indices. All model parameters used to generate artificial behavioral data were successfully estimated by fitting the same models.

*Line 420*

**Fig 3. Results of model recovery in Study 1 (Simulation 2).** A model recovery was conducted to confirm whether the true model showed the best-fit to the behavioral data generated from that model. The artificial data generated by each simulated model were used for model fit. The model with the smallest AIC was adopted as the best fit model for the data. Each model generated 500 sets of artificial data and the data were fitted to all models. The value in each cell indicates the proportion of the best fit model in the 500 sets of artificial data in each simulated model.

*Line 452*

**Fig 4. AIC results after fitting each model to actual behavioral data (Study 1).** * *p* < .05, ** *p* < .001. The black dots, error bars, and colored dots indicate the average, standard deviation (*SD*), and each participant’s data, respectively.

*Line 487*

**Fig 5. The relationships between the degree of conflict and reaction time (RT).** (a) The mean average of RT in high- and low-conflict conditions (back dots), *SD*, and each participant’s data (colored dots). ** *p* < .001. (b) A scatter plot of all the trial data of all the participants between RT and the difference of chosen frequency. The trials with a larger difference in the chosen frequency corresponded to those with less conflict. The black regression line corresponds with all the data points of all the participants. The orange and green dots and the regression lines correspond with the participants’ data that showed the strongest negative correlation and the data that did not, respectively.

*Line 505*

**Fig 6. The relationship between subjective preference rating and chosen frequency in a preference judgment task.** (a) Mean subjective preference ratings for frequently chosen (high-frequency) and less chosen (low-frequency) stimuli (back dots), *SD* (error bars), and each participant’s data (colored dots). (b) A scatter plot of all stimulus data from all participants between subjective preference rating and chosen frequency. The black regression line corresponds to all data points of all participants. The orange and green dots and regression lines corresponding to participant’s data showed the strongest negative correlation and those that did not, respectively.

*Line 624*

**Fig 7. Results of parameter recovery in Study 2 (Simulation 3).** The correlation coefficient between the parameter used to generate artificial data is shown as indices of parameter recovery.

*Line 633*

**Fig 8. Results of model recovery in Study 2 (Simulation 4).** The model with the smallest AIC was adopted as the best-fit model to the data. Each model generated 500 sets of artificial data and the data were fitted to all the models. The value in each cell indicates the proportion of the best-fit model in 500 sets of artificial data in each simulated model.

*Line 647*

**Fig 9. AIC results after fitting each model to actual behavioral data (Study 2).** ** *p* < .001. The black dots, error bars, and colored dots indicate the average, *SD*, and each participant’s data, respectively.

*-------------------------------------------*

Reviewer #4: I am not sure if most researchers in the reward and RL worlds would accept the formulation of IDM. Is it really necessary to build this work on this notion? What if we do not learn the value through a series of decisions, but simply your valuations are noise estimates and by experience we sharpen them? In that case the reward is still external, but we just have to learn the value of the external reward. That seems more general to me, because 0.8 of 1 dollar also has a utility to me personally I need to learn, I know the expected value (0.8) but the utility I have to learn by experience.

*Thank you for pointing that out. As for the distinction between IDM and EDM, a previous meta-analysis study (Nakao et al., 2012) summarized the conceptual and operational differences and reported on the difference of neuronal substrates between those two decision-makings. The difference of neural substrate has also been reported in the other studies (Nakao et al., 2013, 2016, 2019; Wolff et al., 2019).*

*In the IDM domain, the phenomenon called choice-induced preference change (CIPC) has been repeatedly reported in previous studies. It has been assumed that the CIPC reflects the change of value (preference). The original idea of the CBL model was proposed by Akaishi et al. (2014) and Nakao et al. (2016, 2019) who applied the CBL to IDM as the simulation model of CIPC. However, the CBL in IDM has not been tested the appropriateness by fitting the model to the actual behavioral data, and hence we conducted the present study.*

*We realized that we did not clearly describe the previous studies providing the basis for the distinction between IDM and EDM in the original manuscript. We added the following description to the Introduction.*

*-------------------------------------------*

*Line 67*

The IDM and EDM are distinguished conceptually, operationally, and from the neural bases [1,21-24]. Nevertheless, a choice-based learning (CBL) model [18,21,23,25,26] based on RL in EDM has been proposed for the IDM value-learning process. Thus the basic learning principles are assumed to have a commonality between EDM and IDM [18,22,25].

*-------------------------------------------*

*Besides, as the reviewer proposed, it is possible that CIPC reflects the noise reduction by following the preference judgment. In the revised manuscript, we added the model of β changing over time (*T*β*-CBL*) and compared it with the* CBL*ɑ_cr_ model (Model 3 in the original manuscript). Details are as follows.*

*-------------------------------------------*

*Line 566*

# Study 2

In Study 2, we did an exploratory investigation into the possible modification of the CBL model to explain CIPC better. We constructed and validate modified T*β*-CBL (CBL*ɑ_cr_* model with time-varying $\beta$) and F-CBL (CBL*ɑ_cr_* model with the forgetting parameter for unpresented items) based on the CBL*ɑ_cr_* model that had a best fit to the behavioral data in Study 1.

The T*β*-CBL was constructed to examine the possibility that the more a stimulus was experienced, the less noise was included in the IDM. To investigate this possibility, we set th*e β* as a variable over time by following Yechiam et al. [32].

*Line 594*

# Modified CBL models

T$\beta$*-CBL model:* In this model, *β* increases with increasing experiences (i.e., the number of times the items are presented).

$\beta={(\frac{N_{L}^{presented}\left( 1:t \right) + N_{R}^{presented}(1:t)}{2}\cdot\frac{1}{10})^}^{c}$	(9)

$N_{L}^{presented}(1:t)$ and$N_{R}^{presented}(1:t)$ is the number of times the stimuli shown at the left and right sides on the screen are presented until the trial *t. c* is a free parameter that modulates the degree of increase in *β* with experience. The higher the *c* value, the clearer the preference judgment as experience the presentation and judgment of the stimuli.

*Line 619*

# Simulation 3 (Parameter recovery)

Overall, different to Simulation 1, the correlations between the simulated and fitted parameters were not strong in the both the T$\beta$-CBL and F-CBL models (Fig 7, *r*s > .40).

*Line 628*

# Results of Simulation 4 (Model recovery)

Fig 8 shows the mixed matrix resulting from the model recovery. When the T$\beta$-CBL model was the true model, the CBL*α_cr_* model also fitted best in the same proportion as T$\beta$-CBL model. The other two models were able to recover a true model.

*Line 639*

# Results of model fit to the experimental behavioral data

Fig 9 shows a comparison between the models. The result for CBL*α_cr_* is the same as in Fig 4 in Study 1. CBL*α_cr_* model showed better fits to the behavioral data than the other two models (*t*s(47) > 7.55, *p*s < .001; Holm). CBL*α_cr_* showed the best fit for behavior data (*t*s(47) > 2.47, *p*s < .035; Holm). When we compared AIC on an individual participant level, 83.33%, 20.08 %, and 14.58% of the participants showed a best fit to the CBL*α_cr_*, T$\beta$-CBL, and F-CBL models, respectively.

*Line 657*

# Discussion

Study 2 was an exploratory investigation into the possible modification of the CBL model. In the comparison of AIC, the CBL*α_cr_* model showed a better fit to IDM behavioral data than the T$\beta$-CBL and F-CBL models (Fig 9). In contrast, F-CBL showed a better fit in normalized likelihood than CBL*α_cr_* and there was a discrepancy between normalized likelihood and the AIC results. This discrepancy was likely caused by no adjustment for the number of free parameters in normalized likelihood. The F-CBL model had one more free parameter than the CBL*α_cr_* model. The results of Simulation 4 (Fig 8) suggested that if the F-CBL model were the true model, there would be a similar number of participants for whom the CBL*α_cr_* and F-CBL models would be the best fit. In the actual behavioral data results, however, 83.33% of the participants showed a best fit on the CBLacr model. Thus, it could be concluded that the CBL*α_cr_* was a better fit than the other two models in the present study.

In the first place, however, it should be noted that the parameter recovery of the T$\beta$-CBL and F-CBL models was not as good as that of the CBL*α_cr_*. That is, it remains possible that the experimental paradigm of the present study was not designed well enough to validate the two modified CBL models. For example, looking at the results of parameter recovery for the F-CBL model (Fig 7), which was prepared to examine trial-based temporal autocorrelation, adding the forgetting factor *α_F_* in the CBL*α_cr_* model reduced the accuracy of *α_cr_* estimation (c.f., Fig 2). The learning rate *α_cr_* led to a stimulus-based autocorrelation such that the chosen item was more likely to be chosen when presented later. Theoretically, in the present paradigm, the same item was presented every seven trials on average. Hence, it is possible that stimulus-based autocorrelation and trial-based autocorrelation are not easily estimated separately. Future experiments with an increased number of items may be useful to evaluate the F-CBL model properly.

*-------------------------------------------*

In fact I find the setup unexpected. So if I understood this correctly, the introduction claims that this does not work with the usual stimuli that have intrinsic values (like pictures or jobs) that are classically used for the rate-choice-rate task? But instead, this task uses arbitrary shapes. And now the logic is that the choice is the supervision signal to adjust the evaluation? So in a way, this is promoting internal consistency?

*Thank you for your comment. As the reviewer pointed out, the CIPC phenomenon is thought to reflect cognitive processes that lead to promoting internal consistency (Nakao et al., 2016, 2019) that can cause autocorrelation in IDM. The CBL model is one of the computational models representing autocorrelation as a value change in IDM: the chosen stimulus’s value increases and is more likely to be chosen later. Although the CBL model reflects a stimulus-based autocorrelation that does not capture the trial-based temporal autocorrelation since only 2 out of 15 stimuli are presented in each trial. Therefore, we set up an F-CBL that included an attenuation of the value of stimuli not presented. This model will be the best fit in a trial-based temporal autocorrelation in the IDM so that the stimulus chosen in the recent past is more likely to be chosen. Although we examined the trial-based temporal autocorrelation in IDM by comparing this CBL model with the F-CBL model, F-CBL did not show the best fit to the behavioral data. However, the parameter recovery of F-CBL was not as good as the simple CBL models examined in Study 1 in the revised manuscript. That is, it remains possible that the experimental paradigm of the present study was not designed well enough to validate the two modified CBL models. We described this point as a limitation. We added the following descriptions in the revised manuscript.*

*-------------------------------------------*

*Line 137*

# Improvements in CBL-based models

In EDM studies, the RL models incorporating various parameters, such as reducing decision noise with the accumulation of experience in decision-making [32] and value forgetting over time [33], have been proposed. With reference to those EDM studies, as the third aim of the present study, we explored the possible modification of the CBL model to explain CIPC better.

*Line 575*

The F-CBL model was developed to explore the possibility that the value of the items not presented decayed over time. In the CBL models in Study 1, the values of the chosen and the rejected stimuli updated, while the value of the stimulus that was not present in a trial remained unchanged. To examine the possibility of attenuations of the values of unpresented stimuli, we added the forgetting factor ($\alpha_{F}$) to the CBL*ɑ_cr_* model.

The examination of the F-CBL model corresponded to the examination of trial-based temporal autocorrelation in IDM. The CIPC was considered to cause autocorrelation in IDM: chosen items’ preference increased and items were subsequently more likely to be chosen, while rejected items’ preference decreased and items were subsequently less likely to be chosen. The CBL model was one of the computational models that represented such autocorrelation. The CIPC was reflected in the time series behavioral data as a stimulus-based autocorrelation because two out of 15 stimuli were presented in the present study task and that could be captured by CBL*ɑ_cr_* model. However, it was also possible that the item chosen in the recent past was more likely to be chosen, that is, a trial-based temporal autocorrelation. Since the F-CBL included attenuation of the value of stimuli not presented in each trial, this model would be the best fit if there were a trial-based temporal autocorrelation in the IDM.

*Line 602*

*F-CBL model:* In this model, the value *V* of the items *i* that did not presented in each trial *t* was updated as follows:

$V_{i}\left( t+1 \right) ={\left( 1-\alpha_{F} \right)\times V}_{i}\left( t \right)$	$\mathrm{if}i was not presented$	(10)

where $\alpha_{F}$ is the forgetting factor which modulates the degree of attenuation of the value. The forgetting factor was included by following the previous EDM study [33]. In this model, the forgetting factor was applied to unchosen items, unlike the present study.

*Line 670*

In the first place, however, it should be noted that the parameter recovery of the T$\beta$-CBL and F-CBL models was not as good as that of the CBL*α_cr_*. That is, it remains possible that the experimental paradigm of the present study was not designed well enough to validate the two modified CBL models. For example, looking at the results of parameter recovery for the F-CBL model (Fig 7), which was prepared to examine trial-based temporal autocorrelation, adding the forgetting factor *α_F_* in the CBL*α_cr_* model reduced the accuracy of *α_cr_* estimation (c.f., Fig 2). The learning rate *α_cr_* led to a stimulus-based autocorrelation such that the chosen item was more likely to be chosen when presented later. Theoretically, in the present paradigm, the same item was presented every seven trials on average. Hence, it is possible that stimulus-based autocorrelation and trial-based autocorrelation are not easily estimated separately. Future experiments with an increased number of items may be useful to evaluate the F-CBL model properly.

*Line 697*

The third aim was to explore the possible modification of the CBL model. From the computational model analysis of the behavioral data in Study 2, the $\mathrm{CBL}\alpha_{cr}$model fitted better than the other modified models, T*β*-CBL and F-CBL models. However, the parameter recovery of the two modified models was not as good as the $\mathrm{CBL}\alpha_{cr}$model and there remained the possibility that the present study’s paradigm was not appropriate for considering these modifications.

Taken together, it was shown that the CBL$\alpha_{cr}$ model (and also potentially CBL$\alpha_{c}\alpha_{r},$see Discussion in Study 1) could explain CIPC in IDM, the potential for further model improvement remains, though.

*Line 751*

Third, as we discussed in Study 2, to examine the validity of the modified CBL model (i.e., TB-CBL and F-CBL), it is desirable to conduct experiments with appropriate task design to test those models.

*-------------------------------------------*

*The problem that can be avoided by using novel contour shapes is not related to the rate-rate-choice paradigm but is related to the application of a computational model. The initial values affect parameter estimation when using a computational model that assumes that the initial values are equal or use noisy subjective rating as the initial value.
 Although we included rating task after the preference judgment task (choice task), the rating was not used in the computational modeling analysis. The data of choice task was used for the computational model analysis.*

*To clarify those points, we added explanations in the revised manuscripts as follows.*

*-------------------------------------------*

*Line 92*

The first aim of the present study was to examine the appropriateness of the CBL model in IDM by fitting the model to actual behavioral data. We applied the following two strategies to overcome the methodological limitation to applying computation analysis to IDM behavioral data. First, we used preference judgment for novel contour shapes as an IDM task. In EDM studies, novel contour shapes have been used, the initial value of which can be assumed to be equal, and applied to computational model analysis (e.g., [28-30]). By following the EDM studies, we used novel contour shapes to minimize the distortion of model parameter estimation caused by the different initial preferences. Second, we compared the CBL model with a control model without free parameters (e.g., learning rate) in which the probability of choosing an option was estimated based on the chosen frequency in the previous trials. The control model shared the assumption that there was no difference in initial preference among novel contour shapes in the CBL model and preference was estimated based on choice history. Hence, even if the effect of an initial value difference could not be completely ruled out by using novel contour shapes, a better fit of the CBL model to behavioral data than the control model indicated the CBL model’s appropriateness for incorporating free parameter.

*Line 120*

Besides, not using a subjective rating is also useful to avoid pseudo CIPC [12,19], which is caused in cases where both of the following procedures are applied: (1) noise-contaminated subjective ratings to measure CIPC, and (2) calculating the preference changes separately for chosen and rejected items. Although rate-rate choice [12,14,16] and blind choice [13,15] paradigms using subjective rating have been developed to avoid pseudo CIPC, the effect size is small [19]. Developing a method that does not use noise-contaminated subjective rating would lead to a more robust observation of CIPC.

The second aim was to investigate whether the value of both chosen and rejected items changed without using subjective ratings. To achieve this aim, we compared the CBL model with both values changed and with the models with either the value of chosen or rejected item changed. Note that although we collected subjective preference ratings for all novel contour shapes after the IDM (i.e., preference choice) task, the rating data were not for the computational model analysis to examine the CIPC, but to examine the inconsistency between subjective ratings and choice behavior suggested by the previous study [31].

*Line 231*

The data of the preference ratings of the Likert scale were not used in the computational model analysis.

*Line 234*

**Fig 1. Experimental procedure of IDM task and subjective rating.** A) In the IDM task, participants were asked to choose his/her preferred option from two stimuli. The stimuli display time was 2,000 ms. In case participants did not respond during the period, the screen displayed a blank screen until the response. B) In a subjective preference rating, participants subjectively evaluated all 15 stimuli on a 5-point scale (1 = *extremely dislike*, 5 = *extremely like*). The data of the subjective rating were not used for the computational model analysis.

*-------------------------------------------*

In the classic way of studying this, the rate-choose-rate procedure is accompanied by a rate-rate-choose control condition that estimates the baseline amount of increased consistency. How is this model accounting for that effect in the baseline condition? Does it need this control condition too? Presumably the parameter estimates should be smaller in this condition because there cannot be a causal link? (chen and risen 2010 and many papers that cite it). If I understand it correctly in the current study the conditions are just ’choose-rate’ because it is assumed that all shapes are very close in value. It seems to me a rate-choose control condition is necessary?

*As the reviewer described, Chen and Risen (2010) pointed out an important methodological problem in the free-choice paradigm. The paradigm can produce expected preference changes even without any change in true preference (Pseudo-CIPC). Although there are many other ways to prove the existence of CIPC, such as blind choice, rating-rating-choice, and implicit choice, these methods also have their own shortcomings, and the effect size is small (See Izuma’s 2013 research for details). As an option to overcome the problems raised by Chen and Risen, we tested the computational model analysis to examine CIPC in the present study.*

*As the reviewer suggested, the rate-rate-choice paradigm has a control condition for eliminating the possibility of pseudo-CIPC, which is caused in case both of the following procedures are applied: (1) the noise-contaminated subjective ratings were used and (2) the calculating preference changes separately for chosen and rejected items. In the present study’s computational model analysis, we did not use the subjective rating to examine the CIPC. Thus, the present study is not a case in which pseudo-CIPC occurs and we did not include the control condition for controlling the possibility of pseudo-CIPC. We realized that we did not explain this point, and hence we added a description of this in the revised manuscript.*

*Nevertheless, as the reviewer described, we should include a control condition since there is a possibility that the initial value of novel shapes has different initial values. This effect can distort the estimated value of parameters such as learning rate. Hence, we included the control model with an assumption with equal initial values but not a free parameter like CBL models to examine the validity of including free parameters to account for CIPC*

*-------------------------------------------*

*Line 120*

Besides, not using a subjective rating is also useful to avoid pseudo CIPC [12,19], which is caused in cases where both of the following procedures are applied: (1) noise-contaminated subjective ratings to measure CIPC, and (2) calculating the preference changes separately for chosen and rejected items. Although rate-rate choice [12,14,16] and blind choice [13,15] paradigms using subjective rating have been developed to avoid pseudo CIPC, the effect size is small [19]. Developing a method that does not use noise-contaminated subjective rating would lead to a more robust observation of CIPC.

The second aim was to investigate whether the value of both chosen and rejected items changed without using subjective ratings. To achieve this aim, we compared the CBL model with both values changed and with the models with either the value of chosen or rejected item changed. Note that although we collected subjective preference ratings for all novel contour shapes after the IDM (i.e., preference choice) task, the rating data were not for the computational model analysis to examine the CIPC, but to examine the inconsistency between subjective ratings and choice behavior suggested by the previous study [31].

*Line 100*

Second, we compared the CBL model with a control model without free parameters (e.g., learning rate) in which the probability of choosing an option was estimated based on the chosen frequency in the previous trials. The control model shared the assumption that there was no difference in initial preference among novel contour shapes in the CBL model and preference was estimated based on choice history. Hence, even if the effect of an initial value difference could not be completely ruled out by using novel contour shapes, a better fit of the CBL model to behavioral data than the control model indicated the CBL model’s appropriateness for incorporating free parameter.

*-------------------------------------------*

I was surprised that this paper is not discussed ‘Sour grapes and sweet victories: How actions shape preferences’, how the two modelling approaches are related appears to be relevant?

*This is important point. In response to the reviewer’s comment, we added a discussion about the relationship between the research of Vinckier et al (2019) and this study.*

*-------------------------------------------*

*Line 727*

Vinckier et al. [43] used a computational model to examine the cognitive dissonance theory. However, they used an effort task in which participants required effort to get a reward (i.e., foods) and experienced success or failure, and examined the preference change for the foods. The task was closer to EDM than IDM. Although the types of decision-making they addressed were different to those of the present study, it was obvious that preference change did not occur only through simple IDM. Besides, daily decision-making was not simple IDM as focused on in this study, but involved complex factors such as effort, which had been treated in EDM. Future research could consider integrating our model with the models considered by Vinckier et al. [43] and others for an integrated understanding of the human decision-making process.

*-------------------------------------------*

The results in figure 6 are very surprising are they not? The way I understand it, subject chose between items 14 times, but their final appraisals (the ‘rate’ part of the classic rate-choose-rate) shows no trace of this?

*Subjective ratings contain noise (Izuma and Murakami, 2013). It is known that there is a discrepancy between the values reflected in the subjective rating and those actually reflected in choice behavior (Katahira et al., 2011). This can cause the low effect size of CIPC in the studies using both subjective ratings and behavioral choice data (Izuma and Murakami, 2013). Thus, one of the present study’s aims was to examine CIPC from behavioral data without using subjective rating.*

*In previous CIPC studies, a stimulus was presented only once, but in the present study, a stimulus was presented 14 times and chosen several times, as the reviser described. Such multiple choices may lead to discrepancies between the values estimated from behavioral choice and perceived subjective preferences.*

*To clarify those points, we added the following in the revised Discussion*

*-------------------------------------------*

*Line 120*

Besides, not using a subjective rating is also useful to avoid pseudo CIPC [12,19], which is caused in cases where both of the following procedures are applied: (1) noise-contaminated subjective ratings to measure CIPC, and (2) calculating the preference changes separately for chosen and rejected items. Although rate-rate choice [12,14,16] and blind choice [13,15] paradigms using subjective rating have been developed to avoid pseudo CIPC, the effect size is small [19]. Developing a method that does not use noise-contaminated subjective rating would lead to a more robust observation of CIPC.

The second aim was to investigate whether the value of both chosen and rejected items changed without using subjective ratings. To achieve this aim, we compared the CBL model with both values changed and with the models with either the value of chosen or rejected item changed. Note that although we collected subjective preference ratings for all novel contour shapes after the IDM (i.e., preference choice) task, the rating data were not for the computational model analysis to examine the CIPC, but to examine the inconsistency between subjective ratings and choice behavior suggested by the previous study [31].

*Line 552*

The present study’s subjective ratings were not consistent with choice behavior (Fig 6). Subjective ratings were contaminated by rating noise [19]; it is known that there is a discrepancy between the values reflected in the subjective rating and those actually reflected in the choice behavior [31]. This could be the reason for the low effect size [19] in the paradigm of assessing CIPC from a combination of subjective ratings and behavioral choices. That was one of the reasons why we introduced the CBL model analysis to examine CIPC without using subjective ratings. However, it should be noted that the stimuli in the present study were presented 14 times and selected multiple times unlike the typical CIPC experiment [8]. Such multiple selections could lead to large discrepancies between choice behavior (i.e., the chosen frequency in preference judgment) and subjective rating. Therefore, it is important to note that the discrepancy between choice behavioral data and subjective ratings in this study does not negate the paradigm’s usefulness using subjective ratings.

*-------------------------------------------*

Doesn't this mean there is no choice induced chance in the valuation, and it is purely an increased consistency in the choices?

*The possibility that experience in decision-making reduces noise and increases consistency in preference judgments was investigated by introducing the Tβ-CBL model as described above.*

Is there a way of looking at this with higher resolution, by plotting the subjects rating for each of the 15 items to a. The number of times the item was chosen b. The value that the Rl model assigned to that stimulus? Would this results mean that in a classic rate-choose-rate setup, we would conclude that ‘it didn't work’ because there is no effect of choice on rate?

*Since the present study’s paradigm differs from the rate-choice-rate paradigm, the indices cannot be compared directly. Although the subjective rating has been collected in this study, it is not designed for observing CIPC. In this study, CIPC was examined by applying computational modeling to the binary behavioral choice data. Although it is not practical to measure subjective ratings in every trial since it may itself affect the change in preferences, applying a computational model enables us to estimate each trial’s value. We added the explanation about this point in the revised manuscript.*

*-------------------------------------------*

*Line 760*

The computational modeling allowed us to estimate trial-by-trial preference change and model parameters (e.g., learning rate). Those parameters could apply for further analyses, such as for neural bases of IDM, as had been done in EDM research. That would lead to an increase in an integrated understanding of EDM and IDM; for example, whether learning rates, which have common psychological meanings for EDM and IDM, are common in the neural substrates.

*-------------------------------------------*

The discussion mentions two limitations, I am most concerned about the former. It is not really clear to me how to interpret these results and what conclusions to draw from this model, particularly in contrast to existing models. My confusion is increased by the results in figure 6.

*In the present study, the CBL model, in which the value of both selected and unselected stimuli changed, was valid. As the reviewers are concerned, however, the effects of cognitive dissonance need to be examined by constructing the cognitive task to that end. We have added an explanation of this point in the revised manuscript.*

*-------------------------------------------*

*Line 707*

# Cognitive Dissonance Theory

The phenomenon of CIPC has been explained by the theory of cognitive dissonance [43], according to which choosing one item from two items with the same preference subjective rating produces dissonance feelings, which is called “cognitive dissonance.” After that, people adjust their preferences to support their choice as reasonable. That is, they increase their preference for the chosen items and decrease their preference for the rejected items to restore cognitive consistency. Although the present study’s model analysis was not limited to trials with the two items with similar values (i.e., preference), the results supported CIPC. However, by incorporating dissonance into the CBL$\alpha_{cr}$ model it could be a better model to explain CIPC in IDM, and this possibility should be explored in a future study with an appropriate experimental paradigm for that.

*Line 740*

First, the present study showed that the CBL model could describe CIPC by assuming the values of chosen and rejected items are updated as if own choice were the correct answer. However, we did not compare the CBL model with cognitive dissonance theory. To clarify the best model to illustrate CIPC, it would be necessary to compare the CBL model with a computational model that reflected the impact of cognitive dissonance in a future study.

*-------------------------------------------*

Also can you add a URL where the data is in the paper, i couldn't actually make the figshare link work

*Thank you for your comments. I have added the link to the manuscript as follows:*

*-------------------------------------------*

*Line 770*

Please refer to the following link for the raw data: Zhu, Jianhong; Hashimoto, Junya; Katahira, Kentaro; Hirakawa, Makoto; Nakao, Takashi (2020): Computational Modeling of Choice-Induced Preference Change: A Reinforcement-Learning-based approach. figshare dataset. https://doi.org/10.6084/m9.figshare.12083331.v1

*-------------------------------------------*
